# Do pollinators play a role in shaping the essential amino acids found in nectar?

**DOI:** 10.1111/nph.20356

**Published:** 2025-01-02

**Authors:** Rachel H. Parkinson, Eileen F. Power, Kieran Walter, Alex E. McDermott‐Roberts, Jonathan G. Pattrick, Geraldine A. Wright

**Affiliations:** ^1^ Department of Biology University of Oxford Oxford OX1 3SZ UK; ^2^ Department of Botany Trinity College Dublin D02 PN40 Ireland

**Keywords:** *Bombus terrestris*, bumblebee, feeding preferences, methionine, phenylalanine, proline, reward

## Abstract

- Plants produce floral nectar as a reward for pollinators, which contains carbohydrates and amino acids (AAs). We designed experiments to test whether pollinators could exert selection pressure on the profiles of AAs in nectar.
- We used HPLC to measure the free AAs and sugars in the nectar of 102 UK plant species. Six distinct profiles of essential amino acids (EAAs) were defined using the relative proportions of AAs with a clustering algorithm; we then tested bumblebee (*Bombus terrestris*) preferences for the EAA profiles and proline using a two‐choice assay.
- We found a phylogenetic signal for the proportions of phenylalanine, methionine and proline as well as the total concentrations of essential and nonessential amino acids. However, there was no phylogenetic signal for EAA profile. Bumblebees did not exhibit a preference for any of the six EAA nectar profiles; however, four of the EAA profiles stimulated feeding. By contrast, bumblebees avoided proline in an inverse concentration‐dependent manner.
- Our data indicate that bees are likely to have mechanisms for the postingestive evaluation of free AAs in solution but are unlikely to taste EAAs at nectar‐relevant quantities. We predict that EAAs increase nectar value to bumblebees postingestively.

## Introduction

Floral nectar is produced by plants to attract animals to flowers for pollination (Nicolson & Thornburg, 2007). Most floral nectar is dominated by the presence of sugars (mainly sucrose, glucose, and fructose), but the second most abundant metabolites are free amino acids (AAs) (Nicolson & Thornburg, 2007). Free AAs are ubiquitous in nectar, and although quantities can vary considerably (Baker & Baker, 1975; Gottsberger *et al*., 1984; Gardener & Gillman, 2001a; Vandelook *et al*., 2019), they are present at concentrations (micromolar to millimolar) that are substantially lower than the carbohydrates (Nicolson, 2022).

Free AAs in nectar can come from the phloem but can also be produced by the nectary itself (reviewed in Göttlinger & Lohaus, 2024). The concentrations of free AAs in nectar are typically lower than in other plant tissues and may be as much as 100‐fold lower than in the nectaries and phloem (Lohaus & Schwerdtfeger, 2014; Bertazzini & Forlani, 2016; Göttlinger & Lohaus, 2024). While the nectar AA profile (relative abundance of the different AAs) may resemble that in the nectaries (Göttlinger & Lohaus, 2022), it often differs from nectary (Göttlinger & Lohaus, 2024) and phloem (Lohaus & Schwerdtfeger, 2014; Bertazzini & Forlani, 2016) composition, suggesting that the nectar AA profile is more than just a simple filtering of phloem constituents. Some modification of nectar AAs can occur postsecretion through factors such as pollen contamination or microbial action (Peay *et al*., 2012; Bogo *et al*., 2021), though the nectar AA profile could also be under more direct control by plants and driven by pollinator selection (Tiedge & Lohaus, 2017; Göttlinger *et al*., 2019).

Several authors have shown associations between pollinator groups and nectar AA profile and concentration (Baker & Baker, 1975; Petanidou *et al*., 2006; Tiedge & Lohaus, 2017; Göttlinger & Lohaus, 2024); however, others have found that AAs are not as important as other nectar components (Göttlinger *et al*., 2019; Vandelook *et al*., 2019). One criticism of looking for correlations between nectar AAs and pollinator group is that AA concentrations can vary considerably among individuals within a plant species (Gardener & Gillman, 2001a; Gijbels *et al*., 2014). However, large‐scale studies have found that while concentration varies, the relative abundance of individual AAs is much more consistent within species (Gardener & Gillman, 2001a), suggesting AA profile may be a better target for pollinator‐driven selection than absolute concentration. Pollinator discrimination between nectars based on AA composition could reflect metabolic demand (Mevi‐Schütz & Erhardt, 2005) or taste (Gardener & Gillman, 2002).

Insect pollinators, such as bees, rely on pollen as their primary protein source (Wright *et al*., 2018) and only secondarily derive benefits from nectar sources of free AAs (Nicolson, 2022). However, pollinators that do not consume pollen, like some butterflies, may depend more on nectar‐derived AAs (Erhardt & Rusterholz, 1998; Mevi‐Schütz & Erhardt, 2005; Beck, 2007). For this reason, one might predict that bees do not exhibit strong preferences for nectar solutions containing free AAs. However, honeybees have preferences for sugar solutions that contain one of the EAAs over solutions containing one of the nonessential amino acids (NEAAs), with the strongest preference observed towards phenylalanine (Hendriksma & Shafir, 2016), a compound frequently found in floral nectar in high concentrations (Petanidou *et al*., 2006). Other free AAs in sugar solutions, such as the EAA methionine, have been reported to suppress feeding in honeybees (Inouye & Waller, 1984; Simcock *et al*., 2014).

The NEAA proline has attracted specific research attention as it has been found to be the major AA in many nectars (Kaczorowski *et al*., 2005; Carter *et al*., 2006; Terrab *et al*., 2007) and is also the dominant AA in the haemolymph of both honeybees (Crailsheim & Leonhard, 1997) and bumblebees (Stabler *et al*., 2015). Proline can also act as a substrate for powering insect flight (Bursell, 1975; Auerswald & Gäde, 1999), though evidence for this role in bees is equivocal. In honeybees, haemolymph proline concentration decreases significantly after flight (Barker & Lehner, 1972; Micheu *et al*., 2000), though the proportional contribution compared with carbohydrates is minimal (Barker & Lehner, 1972). Isolated flight muscles from *Bombus impatiens* bumblebees showed significant increases in the rate of respiration when exposed to proline (Teulier *et al*., 2016); however, this effect was not found with mitochondria from males of the bumblebee *B. terrestris* (Syromyatnikov *et al*., 2013). More recent work with whole living bumblebees suggests that, at least in *Bombus impatiens*, proline is most likely used as a sparker for carbohydrate metabolism for the first few minutes of flight (Stec *et al*., 2021). Honeybees appear to prefer sucrose solutions containing 2–10 mM proline concentrations (Carter *et al*., 2006; Bertazzini *et al*., 2010) but find higher proline concentrations aversive (Carter *et al*., 2006; Simcock *et al*., 2014). Other bee species may have different preferences, however. One study reported that nectar‐relevant proline of *c*. 2 mM appeared to suppress sugar solution consumption in bumblebees (Bogo *et al*., 2024). While many studies have examined individual AAs in nectar, few have reported how the profiles or mixtures of AAs found naturally occurring in nectar influence the preferences of pollinators.

Although nectar AAs clearly impact the feeding behaviour of bees, we know little about how they detect these compounds (de Brito Sanchez, 2011; Stabler *et al*., 2015). There is evidence that *B. terrestris* can detect but cannot discriminate between multiple AAs using antennal sensory receptors (Ruedenauer *et al*., 2019) and that gustatory receptors on the mouthparts of honeybees respond to AAs (Lim *et al*., 2019). However, both these studies used AA concentrations much higher than those typically found in nectar. Even if bees are unable to detect AAs by contact chemosensation, effects on feeding behaviour may still occur through postingestive mechanisms (Stabler *et al*., 2015).

In this study, we analysed the carbohydrate and AA profiles of nectar from 102 plant species from the UK and tested whether these metabolites reflected phylogenetic relationships among the selected plant species. Based on previous work where we observed that the EAAs were the most important components of protein for bumblebees (Stabler *et al*., 2015), we examined the impact of EAAs on bumblebee preferences for nectar. We identified six distinct essential amino acid (EAA) profiles in UK plant species nectar and tested the preferences of buff‐tailed bumblebees for these nectar profiles. We also tested bumblebee preferences for proline, a NEAA found in some nectars at high concentrations. We consider our results from the context of pollinators potentially exerting selective pressure on nectar AA composition, highlighting the importance of the EAA profile rather than absolute concentrations, and how EAAs may increase nectar value in a way that is independent of sensory cues.

## Materials and Methods

### Nectar data collection

Nectar data were collected from 102 species in 2011 and 2012 from 56 unique sites in north‐eastern and southern England. We sampled nectar using 1 μl microcapillary tubes (Hirschmann Laborgeräte GmbH & Co. KG, Eberstadt, Germany) to extract raw nectar from open, unbagged, flowers with healthy appearances (no signs of senescence). The microcapillary method minimises potential contamination during the sampling process from for example pollen or vascular fluid (Power *et al*., 2018). We sampled from sufficient flowers to ensure sufficient nectar volume for chemical analysis. The number of flowers per sample therefore depended on the nectar volume per flower and varied between an average of 1 and 35 flowers (Supporting Information Table S1). Nectar samples were kept in a cool bag containing an ice block until return to the laboratory where they were stored in a freezer (*c*. −20°C). Before analysis, nectar samples were diluted with UHPLC gradient grade water (Fisher Scientific UK Ltd, Loughborough, UK) to meet minimal sample volume requirements for carbohydrate and AA analyses (as in Power *et al.* 2018). Samples were centrifuged to remove any residual plant material.

### Nectar sample analysis

Nectar sugar composition (sucrose, glucose, and fructose) was analysed using high performance ion chromatography (HPIC). For each sample, *c*. 30 μl of diluted nectar was inserted into an analysis vial to ensure optimal immersion of the autosampler syringe, and 20 μl of this nectar sample was injected via a Rheodyne valve onto a Carbopac PA‐100 column (Dionex, Sunnyvale, CA, USA) fitted with a Dionex Carbopac PA‐100 BioLC guard (4 × 50 mm). Sample components were eluted isocratically using 100 mM NaOH (de‐gassed by helium gas) flowing at 1 ml min^−1^ for 10 min at room temperature (RT).

We recorded the chromatographic profiles using pulsed amperometric detection with an ED40 electrochemical detector (Dionex, Sunnyvale, CA, USA) and analysed the elution profiles using chromeleon v.6.8 (Thermo Fisher Scientific Inc., Waltham, MA, USA). The HPIC was calibrated at least twice every 24‐h period for all compounds of interest by injecting calibration standards with concentrations of 10 ppm each. Standard solutions were made from the solid forms of each sugar (Sigma‐Aldrich). Measured concentrations were rescaled to the original concentration using the dilution factor.

Amino acid composition was determined using ultra high‐performance liquid chromatography (uHPLC). This gave the concentrations of all 20 standard protein‐forming AAs and GABA. We used an automated precolumn derivatisation programme for the autosampler (Ultimate 3000 Autosampler, Dionex, Thermo Fisher Scientific Inc.) immediately before injection to pretreat 10 μl of sample. This was comprised of: 1 min with 15 μl of 7.5 mM o‐phthaldialdehyde (OPA) and 225 mM 3‐mercaptopropionic acid (MPA) in 0.1 M sodium tetraborate decahydrate (Na_2_B_4_O_7_.10H_2_O), pH 10.2; 1 min with 10 μl of 96.6 mM 9‐fluroenylmethoxycarbonyl chloride (FMOC) in 1 M acetonitrile, followed by the addition of 6 μl of 1 M acetic acid. After pretreatment, 30 μl of the AA derivatives were injected onto a 150 × 2.1 mm Accucore RP‐MS uHPLC‐column (Thermo Fisher Scientific Inc). Elution solvents were: *A* = 10 mM di‐sodium hydrogen orthophosphate (Na_2_HPO_4_), 10 mM Na_2_B_4_O_7_.10H_2_O, 0.5 mM sodium azide (NaN_3_), adjusted to pH 7.8 with concentrated HCl, and *B* = Acetonitrile : Methanol : Water (45 : 45 : 10 v/v/v). Elution of the column occurred at a constant flow rate of 500 μl min^−1^ using a linear gradient of 3 to 57% (v/v) of solvent B over 14 min, followed by 100% solvent B for 2 min, and a reduction to 97% solvent B for the remaining 4 min (Power *et al*., 2018).

The derivatives were detected by fluorescence (Ultimate 3000 RS Fluorescence Detector, Dionex, Thermo Fisher Scientific, OPA: excitation at 330 nm and emission at 450 nm, FMOC: excitation at 266 nm, and emission at 305 nm) and quantified by automatic integration after calibration of the system with AA standards. Reference calibrations (for all AAs) were conducted following the processing of each batch of 20 samples by injecting calibration standards (Sigma‐Aldrich). Elution profiles were analysed using chromeleon software v.6.8 (Thermo Fisher Scientific Inc). Amino acid peaks were detected automatically based on precalibrated elution times, with all peaks checked to ensure correct identification.

### Nectar dataset analyses

Concentrations of nectar sugars were adjusted based on sucrose equivalency, such that for glucose and fructose 1 M = 0.5 sucrose‐equivalent molarity (se‐M, Fleming *et al*., 2008; Leseigneur & Nicolson, 2009; Pattrick *et al*., 2024). We summed the se‐M concentrations to obtain a measure of total sugars and calculated the proportion of the total represented by each sugar. For AAs, the proportions of the EAAs (Arg, arginine; His, histidine; Ile, isoleucine; Leu, leucine; Lys, lysine; Met, methionine; Phe, phenylalanine; Thr, threonine; Trp, tryptophan; Val, valine) and NEAAs (Ala, alanine; Asn, asparagine; Asp, aspartic acid; Cys, cystine; Gln, glutamine; Glu, glutamic acid; Gly, glycine; Pro, proline; Ser, serine; Tyr, tyrosine; and the nonprotein‐forming gamma‐aminobutyric acid; GABA) were calculated as proportions of the total AAs (the summed concentrations of all essential and NEAAs). Data were averaged across samples for each species. Amino acids were classified as essential vs nonessential based on data from honeybees (de Groot, 1953).

To visualise the high‐dimensional relationships between nectar EAA profiles across species, we employed t‐distributed stochastic neighbour embedding (t‐SNE). We standardised (*z*‐scored) the EAA proportion data, and the perplexity parameter was set to 20 to balance local and global aspects of the data. We determined the optimal number of clusters in the t‐SNE reduced‐dimensional space with a silhouette analysis, which measures the cohesion and separation of clusters. The average silhouette width was calculated for *k* = 2 to 20, and the number of clusters with the highest silhouette width was selected as optimal. Using this optimal k, we applied K‐means clustering to the t‐SNE coordinates. We reconstructed the proportions of each EAA (relative to total AAs) and sucrose‐equivalent sugar proportions for sucrose, glucose and fructose from the nectar clusters. The mean nectar concentrations of EAAs and sugars were also reconstructed from the species in each cluster.

We assembled a phylogenetic tree by pruning an existing dated tree with the species from our dataset (Zu *et al*., 2021). Species from our dataset that were not present in the template tree were either replaced by another species from the same genus in the template tree (if the missing species was the only representative of a genus) or otherwise removed.

### Preference assays

We assessed the preferences of bumblebees (*Bombus terrestris audax* (Harris, 1776)) for sugar mixtures with or without AAs using a caged preference assay (Tiedeken *et al*., 2014). Queenright bumblebee colonies, containing *c*. 100–200 individuals, were reared by Biobest (Westerlo, Belgium), purchased from Agralan (Ashton Keynes, UK), and maintained at RT in their colony boxes with access to Biogluc (Biobest) and provided with honeybee‐collected mixed floral freeze‐dried pollen (Agralan, Swindon, UK). For the preference assays, female worker bees were caught directly from colonies. Very small bees were excluded from the assay following visual inspection. Individual worker bees were assigned to treatment groups such that at least two colonies were tested in every group. Bees were housed in groups of five per cage and provided with *ad libitum* access to the test solutions from four Eppendorf tubes modified with four 2 mm drinking holes along one side. Tubes were affixed to the cages (two on each side) and balanced within treatment groups. These cages were kept in incubators at 28°C, 60% relative humidity, in darkness, for 72 h in total for each assay. Pollen was not provided during the assay.

We performed three separate preference assays to test the bees' preferences for EAAs and proline: (1) comparing the sugar backgrounds with and without the mean nectar EAA concentrations for each of the six clusters identified from the clustering analysis (Tables 1, S2); (2) a concentration gradient of cluster B EAA (from 1 to 1000 μM) and sugar profiles vs the sugar background alone, selected due to this cluster having the highest nectar EAA concentrations (Table S2); and (3) a concentration gradient of proline (1 to 1000 μM) in the sugar background vs the sugar background containing 0.5 se‐M each of glucose and fructose (Table S2). Test solutions were prepared with reagent‐grade sucrose, glucose, fructose, EAAs, and proline (Merck Life Science, Arklow, UK).

**Table 1:** Amino acid profiles of clusters from nectars dataset.

	Cluster A	Cluster B	Cluster C	Cluster D	Cluster E	Cluster F
Total EAA (μM)	0.10	21.42	1.53	2.33	12.10	8.22
His (μM)	0.00	0.75	0.13	0.01	0.69	0.55
Thr (μM)	0.00	1.00	0.05	0.28	0.32	0.23
Arg (μM)	0.00	2.15	0.07	1.15	0.68	0.75
Val (μM)	0.00	1.53	0.11	0.43	1.17	3.06
Met (μM)	0.04	6.40	0.49	0.01	0.58	0.87
Trp (μM)	0.00	0.44	0.00	0.01	0.00	0.00
Phe (μM)	0.01	3.22	0.19	0.14	7.45	0.91
Ile (μM)	0.00	0.68	0.11	0.14	0.50	0.61
Leu (μM)	0.00	1.09	0.26	0.13	0.49	0.76
Lys (μM)	0.04	4.15	0.12	0.04	0.22	0.49
Total sugars (seM)	0.65	0.71	0.79	0.59	0.72	0.67
Sucrose (seM)	0.21	0.25	0.33	0.18	0.29	0.26
Fructose (seM)	0.24	0.28	0.28	0.25	0.30	0.25
Glucose (seM)	0.21	0.18	0.19	0.15	0.14	0.16

Values are given as mean concentrations for each cluster, with sugar concentrations reported as sucrose‐equivalent molarity (seM). Grey shading shows total essential amino acid (EAA) and sugar concentrations for each solution.

The assays ran for 72 h (*n* = 15 cages per group) and split into three blocks of 24 h. During the first 24 h bees were acclimated to the feeding cages and provided with the background sugar solutions only (no AA solutions). For the second, 24‐h block Eppendorf tubes were replaced with tubes containing fresh solutions (still background sugars only). Consumption data from this second block was used as a no‐choice control to demonstrate whether the bees preferred to drink from either side of the cage and obtain baseline consumption volumes. For the final 24 h, tubes were again replaced but bees were given a choice between the sugar solution, or a solution containing EAAs in the same sugar background. Evaporation was controlled for on each day by including five replicates of tubes for each solution in cages with no bees.

We calculated the volume consumed by weighing the solutions before and after each 24‐h period, with the average evaporation quantities taken from the totals and then converted to volume using the density of the solutions. A preference index was calculated using the following formula:

$$\frac{\text{volume of test solution}\;-\;\text{volume of control solution}}{\text{volume of test solution}+\text{volume of control solution}}$$

We recorded any mortality on each day of the experiment and scaled the average consumption per bee by the number of surviving individuals. Following each assay, bees were euthanised by freezing at −20°C.

To estimate the proportion of the 102 plant species that are visited by bumblebees, we used The Database of Pollinator Interactions (Balfour *et al*., 2022). A species was classed as visited by *Bombus terrestris/lucorum* if there were any recorded interactions from bees in these taxa. *Bombus terrestris* and *Bombus lucorum* workers are indistinguishable in the field and so are typically grouped together.

### Statistical analyses

All statistical analyses were performed in R v.4.3.3 (R Core Team, 2024). Data were tested for normality with Shapiro–Wilk tests. Data that were not normally distributed were transformed or tested with nonparametric approaches. We assessed whether the average concentrations of AAs (log_10_ concentrations of EAAs and proline) or sugars varied across species using nonparametric Friedman tests, accounting for repeated measures within species. Pairwise comparisons were conducted using the Nemenyi–Wilcoxon–Wilcox all‐pairs *post hoc* test, with a single‐step *P*‐value adjustment for multiple comparisons. Amino acid proportion data was logit transformed, following which we explored the correlation between the average proportions of EAAs and proline across species using Pearson's correlation coefficients, retaining only significant correlations (*P* < 0.05) for interpretation and visualisation.

We compared AA and sugar profiles across cluster assignments using PERMANOVA with adonis *post hoc* multiple comparisons performed with the vegan package (Oksanen *et al*., 2024). We fitted beta regression generalised mixed effects models (GLMM) with a logit link function to assess differences in the proportions of AAs and sugars within each cluster, accounting for repeated measures by including the species as a random effect. The models were fitted using the glmmtmb package in R (Brooks *et al*., 2017). We assessed which AAs represented the largest proportions in each cluster by comparing the marginal means with the overall mean using the emmeans package v.1.10.5 (Russell *et al*., 2021). We assessed the phylogenetic signal of the nectar profiles (the two dimensions from the t‐SNE analysis, individual AA proportions, total AA concentrations, sugar concentrations, and sugar proportions) with Blomberg's K and Pagel's Lambda (Pagel, 1999; Blomberg *et al*., 2003) using the phylosignal package in R (Keck *et al*., 2016).

For the behavioural data, we modelled the preference indices and total volume consumed using linear mixed effects (LME) models comparing between the sugar only (no choice) and test days, as well as across groups, with cage ID as the random effects using the lmertest package in R (Kuznetsova *et al*., 2017). *Post hoc* tests were performed with the emmeans package, with *P*‐values adjusted for multiple comparisons using the Tukey method. Mortality was assessed over the duration of each preference assay using Kaplan–Meier survival analysis. Exact *P*‐values are provided unless *P* < 0.0001.

## Results

### Nectar amino acid and sugar concentrations

**Fig. 1:**
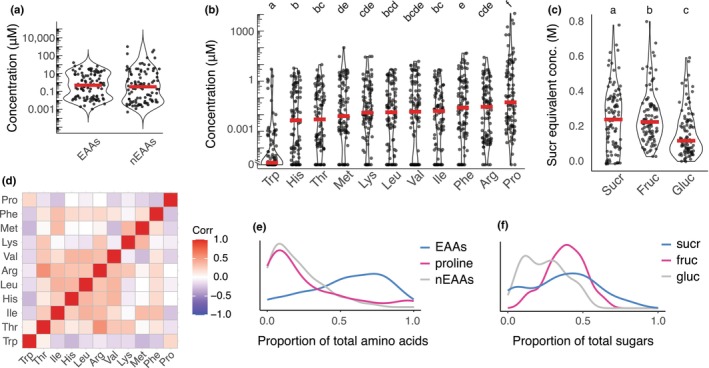
Amino acids and nectar sugars from 102 plant species. (a) Concentrations of essential amino acids (EAAs) and nonessential amino acids (nEAAs) for all species (*n* = 102). Violin plots with individual datapoints represent average concentrations per species (*n* = 102 species, min = 3, max = 28 samples/species), and medians as red lines. (b) Nectar concentrations of EAAs and proline (Friedman χ^2^ = 277, df = 10, *P* < 0.0001, *n* = 102). Significant differences between groups (Nemenyi–Wilcoxon–Wilcox *post hoc*) are denoted with letters. Violin plots with datapoints represent average concentrations per species and medians as red lines. (c) Sucrose‐equivalent nectar concentrations of sucrose, fructose, and glucose (Friedman χ^2^ = 51.8, df = 2, *P* < 0.0001, *n* = 102). Significant differences between groups (Nemenyi–Wilcoxon–Wilcox *post hoc*) are denoted with letters. Violin plots with datapoints represent average concentrations per species and medians as red lines. (d) Pearson correlation coefficients of the logit‐transformed proportions of EAAs and proline across all species in the dataset. Only significant correlations are plotted (nonsignificant correlations are set to 0 and plotted in white). (e) The distribution of the average EAAs, proline, and other nEAAs as a proportion of total amino acids across all species. (f) Distribution of the average sucrose‐equivalent concentrations of sucrose (sucr), fructose (fruc), and glucose (gluc) across all species.

Our nectar dataset included 102 plant species, 54% of which are known to be pollinated by *Bombus terrestris*, and 58% by other bumblebee species (Balfour *et al*., 2022; Table S1). However, 88% of the genera included have at least one species pollinated by bumblebees (Balfour *et al*., 2022; Table S1). The nectar showed substantial variation in total EAA concentration (median = 0.49 μM, IQR = 8.45 μM) and NEAA concentration (mean = 0.33 μM, IQR = 10.61 μM), with a range spanning nanomolar to millimolar concentrations across species (Fig. 1a). The concentrations of individual AAs also varied considerably (Fig. 1b). Trp exhibited the lowest mean concentration in nectar (mean = 0.113 μM; SE = 0.44 μM), whereas Pro (mean = 217 μM, SE = 152.80 μM) was almost always present at higher concentrations than any of the AAs. The most extreme value for proline was observed in the nectar of *Iris pseudacorus* (mean = 11.4 mM).

We used an AA concentration cut‐off of 0.001 μM to quantify which AAs were represented in the species in our nectar dataset. Of all the AAs, Pro was found in the highest proportion of species (87% of species). Of the other NEAAs, Asn, GABA, and Gln were present in only *c*. 20% of species, while Asp, Glu, Ser, Gly, Ala, Tyr, and Cys were present in *c*. 60% of species. Each of the EAAs was present in between 54% (His and Thr) and 76% (Phe) of species, except for Trp, which was found in just 17% of species at concentrations > 0.001 μM. When looking at the number of EAAs found in individual species, 32% of species had fewer than five EAAs, while 47% had between eight and 10 EAAs. Patterns were similar for NEAAs, with 40% of species having fewer than five NEAAs, and 42% with between 8 and 11 NEAAs.

The three nectar sugars also varied, with significantly lower sucrose‐equivalent molarity (se‐M) concentrations of glucose (median = 0.13 M, SE = 0.03 M) compared with fructose (median = 0.24 M, SE = 0.06 M) or sucrose (mean = 0.25 M, SE = 0.07 M; Fig. 1c). The proportions of some individual EAAs + Pro were correlated across samples, with the highest positive correlation between Arg and Thr, and the largest negative correlation between Met and Trp (Fig. 1d). On average, EAAs represented a greater proportion of the total AAs, but some species had nectar containing almost exclusively proline (Fig. 1e). Fructose was the dominant nectar sugar in most species, and no species had glucose‐dominated nectar (Fig. 1f).

### Clustering analysis of essential amino acid profiles

**Fig. 2:**
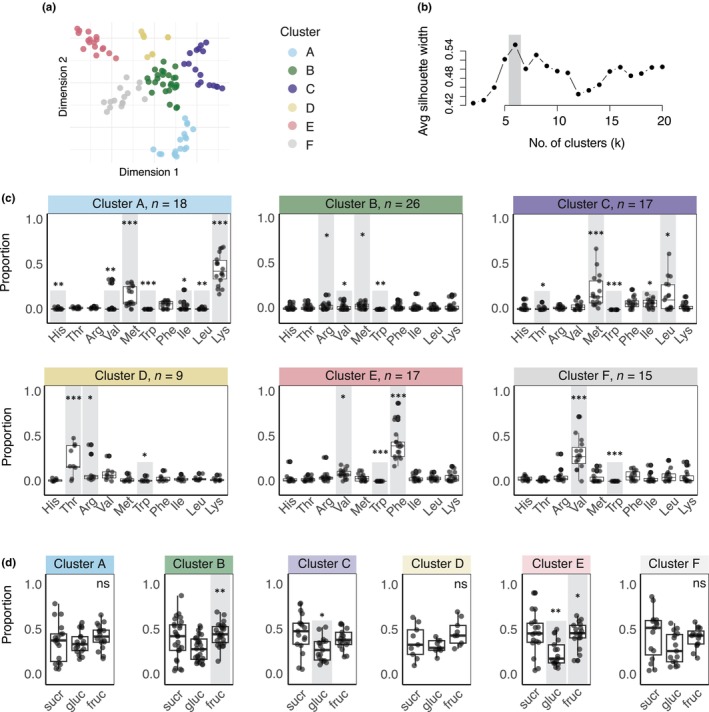
Clustering of the proportion of total amino acids represented by each essential amino acid (EAA). (a) t‐distributed stochastic neighbour embedding (t‐SNE) dimensionality reduction in the EAA proportion data. Each point represents a species, and the distance between points reflects the similarity of EAA profiles (*n* = 102). Clustering with k‐means revealed six groupings (coloured) based on EAAs. (b) Silhouette plot showing silhouette widths against the number of clusters. *K* = 6 was identified as the optimal cluster number with the highest silhouette width. (c) EAA profiles varied significantly across clusters (PERMANOVA: *F*_5_ = 27.6, *R* ^2^ = 0.59, *P* = 0.001), with significant differences in amino acid profiles between each cluster (adonis pairwise multiple comparisons, adjusted *P* = 0.015). Within each cluster, the relative proportions of EAAs varied (generalised mixed effects models (GLMM), cluster A: χ^2^ = 330, df = 9, *P* < 0.0001; cluster B: χ^2^ = 52.6, df = 9, *P* < 0.0001; cluster C: χ^2^ = 89.6, df = 9, *P* < 0.0001; cluster D: χ^2^ = 53.0, df = 9, *P* < 0.0001; cluster E: χ^2^ = 344, df = 9, *P* < 0.0001; cluster F: χ^2^ = 94.1, df = 1, *P* < 0.0001). Amino acids with significantly higher or lower estimated marginal means compared with the overall mean of the cluster are highlighted with long or short grey bars, respectively, with asterisks denoting the significance of these *post hoc* tests (emmeans). Boxplots show median and interquartile range. (d) Sugar profiles did not vary significantly across clusters (PERMANOVA: *F*_5_ = 0.805, *R* ^2^ = 0.0402, *P* = 0.59). Within cluster, there were significant differences between the proportions of sugars for clusters B, C, and E (GLMM, cluster B: χ^2^ = 9.79, df = 2, *P* = 0.0075; cluster C: χ^2^ = 6.19, df = 2, *P* = 0.045; cluster E: χ^2^ = 10.6, df = 2, *P* = 0.0049). There were no significant differences between the proportions of sugars in Clusters A, D, and F (cluster A: χ^2^ = 3.48, df = 2, *P* = 0.18; cluster D: χ^2^ = 5.77, df = 2, *P* = 0.056; F: χ^2^ = 2.90, df = 2, *P* = 0.23). Sugars with significantly higher or lower estimated marginal means compared with the overall mean of the cluster are highlighted with long or short grey bars, respectively, with asterisks denoting the significance of these *post hoc* tests (emmeans). Boxplots show median and interquartile range. ns, not significant.

A clustering analysis using the profile of EAAs in nectar (Fig. 2a,b) revealed six distinct nectar types (PERMANOVA: *F*_5_ = 27.6, *R* ^2^ = 0.59, *P* = 0.001) driven by the proportions of individual EAAs (Fig. 2c). Within each cluster, one or two EAAs were present at significantly higher proportions than the other EAAs: for example, Met and Lys (cluster A), Arg and Met (cluster B), Met and Leu (cluster C), Thr and Arg (cluster D), Val and Phe (cluster E), and Val (cluster F).

The clusters also displayed distinct mean total concentrations of EAAs (Table 1), ranging from 0.1 μM (cluster A) to 21.4 μM (cluster B). The proportions of nectar sugars, however, did not vary significantly across clusters (PERMANOVA: *F*_5_ = 0.805, *R* ^2^ = 0.0402, *P* = 0.59), although there were differences in the proportions of sugars within some of the clusters (Fig. 2d; Table 1).

We compared whether the cluster assignments were predictive of whether the species of plants were known to be pollinated by bumblebees (Balfour *et al*., 2022). There were differences in the proportions of species associated with *Bombus terrestris*: only 33% of the species in cluster A are known to be visited by *Bombus terrestris*, while 78% of the species in cluster B have been associated with *Bombus terrestris* visitors (Balfour *et al*., 2022); however, the difference in proportions between clusters was not significant (GLM, χ^2^ = 5.86, df = 5, *P* = 0.320).

### Phylogenetic signal in nectar composition

**Fig. 3:**
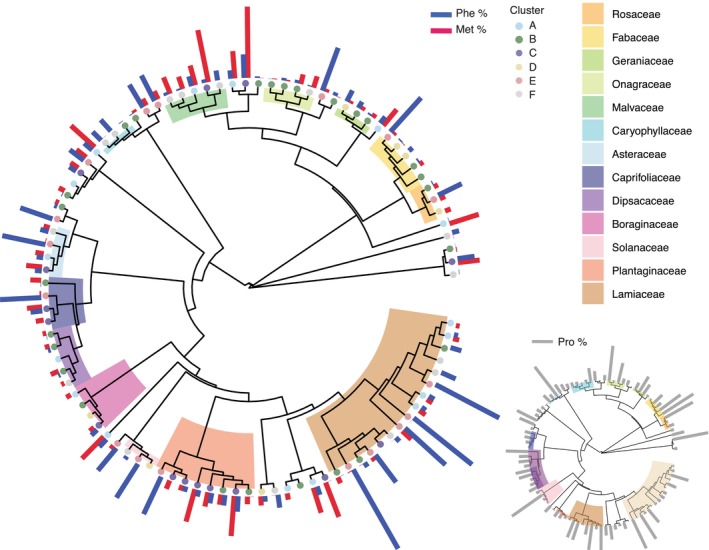
Phylogenetic analysis of amino acid profiles. Dated phylogenetic tree containing 96 species from our nectar dataset (pruned from Zu *et al*., 2021). Families with at least three representative species are labelled with coloured blocks. Tip point circles show the cluster assignment based on the proportions of essential amino acids (EAAs). Bars show the proportions of phenylalanine (Phe %) and methionine (Met %) for each species, as these EAAs had a phylogenetic signal. The inset phylogenetic tree shows the proportion of proline (Pro %) across species, which was the only non‐EAA with a phylogenetic signal.

The presence of phylogenetic signal was quantified using Blomberg's K and Pagel's Lambda (Pagel, 1999; Blomberg *et al*., 2003). These analyses revealed significant phylogenetic signal across several measures, most notably for the total concentration of EAAs, where there was a strong signal (λ = 0.913, *P* = 0.001, *K* = 0.44, *P* = 0.009; Fig. S1; Table S3). We also found weaker signal for the total concentration of NEAAs (Fig. S1a), for the proportions of Met, Phe, and Pro (Fig. 3; Table S3) and the proportions of glucose and sucrose in nectar (Fig. S1b); though each of these parameters were only significant from one of the two indices. Pagel's Lambda was significant for the proportion of Met, indicating that closely related species have more similar Met profiles. By contrast, Blomberg's K was significant for the proportions of Pro and Phe. None of the other EAAs showed any significant phylogenetic signal (Table S3). Despite the strong signal for the total concentration of EAAs, we found negligible signal for the profile of EAAs (as quantified by the t‐SNE dimensionality reduction). This suggests that while total AA concentration and individual EAAs may be determined by phylogeny, the overall blend of AAs may be driven by other factors such as pollinator preference.

### Bumblebees drink more when EAAs are present in feeding choices

**Fig. 4:**
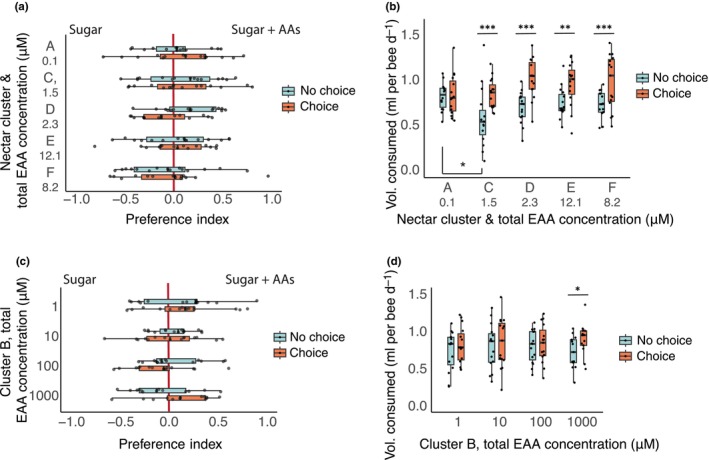
Bumblebee (*Bombus terrestris*) preferences for sugar solutions with nectar‐relevant amino acid profiles. (a) Preference assay using sugar and amino acid profiles from clustering analysis at nectar‐relevant concentrations (see Table 1, *n* = 15 cages per group). Total essential amino acid (EAA) concentrations for each cluster are shown on the y‐axis. There was no significant effect of adding EAAs on preferences (LME: choice, *F*_1,143_ = 0.443, *P* = 0.51; cluster, *F*_4,143_ = 0.826, *P* = 0.51). Boxplots show the median and interquartile range of preferences on acclimation day (no choice) and test day (choice), with points representing individual cages. (b) Total volume consumed (summing sugar‐only and amino acid solutions from A, *n* = 15 cages per group). Significant interaction between day and nectar group (LME, day*group: *F*_4,69_ = 3.36, *P* = 0.014, day: *F*_1,69_ = 52.8, *P* < 0.0001; nectar group: *F*_4,69_ = 1.76, *P* = 0.15). Boxplots show the median and interquartile range. Asterisks indicate significant *post hoc* comparisons (emmeans, Tukey adjustment). (c) Preference assay comparing sugar solutions vs a concentration gradient of the Cluster B EAA profile (1 to 1000 μM, *n* = 15 cages per group). There were no significant effects of EAAs on preferences (LME, day: *F*_1,54_ = 0.0686, *P* = 0.79, EAA group: *F*_3,54_ = 1.19, *P* = 0.32). Boxplots show the median and interquartile range of acclimation (no choice) and test day (choice) preferences, with points representing individual cages. (d) Total volume consumed from C (*n* = 15 cages per group). There were significant differences between acclimation (no choice) and test (choice) days (LME: day, *F*_1,54_ = 8.44, *P* = 0.0053; group, *F*_3,54_ = 0.103, *P* = 0.96; day*group, *F*_3,54_ = 0.959, *P* = 0.42). Boxplots show the median and interquartile range. Asterisks indicate significant *post hoc* comparisons (emmeans, Tukey adjustment).

We created nectar solutions to match the mean concentrations of each EAA and sugar from the nectar profiles (Tables 1, S2). In all cases, the bees did not show a preference for nectar sugar mixtures containing EAAs over the sugar mixtures alone (Fig. 4a), indicating that EAA profiles did not drive an aversion to or a preference for nectar. However, if the total volume of solution consumed during the test period is considered, we found that for four out of the six nectar solutions, the bees consumed more food overall when EAAs were present in one of the solutions at nectar‐relevant concentrations (Fig. 4b).

The cluster B AA profile was tested over a concentration gradient from 1 to 1000 μM total EAAs (Table S2). This cluster had the highest mean nectar concentrations of EAAs and included every EAA at concentrations between 0.44 μM (Trp) and 6.40 μM (Met). The concentration of the cluster B EAA nectar profile did not affect the bees' preference over sugar alone (Fig. 4c). However, only at the highest concentration we tested were we able to observe that EAAs affected the total volume of all food consumed in the experiment (Fig. 4d). There was no significant effect of any of the EAA mixtures on survival (Fig. S2a,b).

### Bumblebees avoid drinking proline

**Fig. 5:**
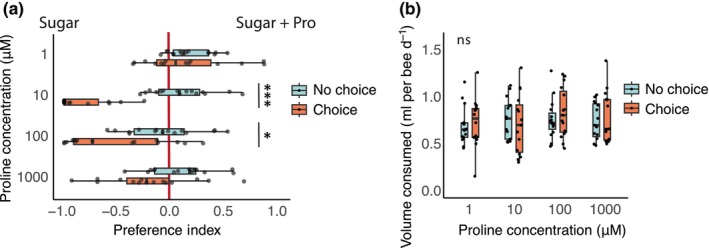
Bumblebee (*Bombus terrestris*) preferences for sugar solutions containing proline. (a) Preference assay comparing the preferences of bumblebees to sugar mixtures with or without proline (1–1000 μM, *n* = 15 cages per group). Individual cages were tested with a no choice (sugar only) control day preceding the test day. Boxplots show median and interquartile range, with individual data points plotted for each cage. Asterisks show significant differences between no choice and test day (emmeans, Tukey adjustment). (b) Total volume consumed (sugar solution and sugar + proline solution) on the no‐choice and choice days respectively (*n* = 15 cages per group). Boxplots show median and interquartile range, with individual data points plotted for each cage. There was no significant (ns) effect of the choice on proline consumption (emmeans, Tukey adjustment).

Bees exhibited a weak preference for solutions containing 1 μM Pro, but in general, we found that bumblebees avoided drinking solutions containing Pro over concentrations from 10 to 100 μM (Fig. 5a; Table S2). There was no significant effect of Pro on the total volume of food consumed (Fig. 5b), and none of the Pro solutions significantly affected survival over the duration of the assay (Fig. S2c).

## Discussion

The presence of AAs in nectar has been known for over 50 yr (Baker & Baker, 1973), but whether they are a trait under selection by pollinators is poorly understood (Parachnowitsch *et al*., 2019). Here, we investigated the importance of AAs for pollinators by focussing on the relative proportions of the EAAs in nectar. We report evidence that while overall AA concentrations and the proportions of some AAs (Pro, Phe, and Met) of the nectar of UK plant species are phylogenetically conserved, variation in the complete profile of nectar EAAs is a much more divergent trait. In general, nectar EAAs increased feeding behaviour of bumblebees. Together, this suggests that the blend of AAs in nectar may be a target for pollinator‐driven selection.

Using the EAA profile (proportions of individual EAAs relative to the total AA concentration), the nectars clustered into six groups; in each case, these profiles were characterised by one or two prominent EAAs. When we tested the nectar of these six groups on bumblebees, the bees did not prefer or avoid any of the solutions, indicating that they are unlikely to taste these AAs at micromolar concentrations. However, with some EAA profiles, the bees ate significantly more food overall. This suggests that low levels of EAAs in food provide a postingestive signal of value that drives feeding. This effect was found for four out of the six nectar EAA mixtures at nectar‐relevant concentrations. By contrast, the NEAA Pro was generally avoided at concentrations > 1 μM but its presence did not affect the total amount of food eaten by the bees.

Unlike other floral traits such as colour or scent, there has been comparatively little research on the potential for pollinators to act as selective agents on nectar chemistry (Gijbels *et al*., 2015b). For example, few other studies have quantified the degree of phylogenetic signal on nectar AAs (Gijbels *et al*., 2015b). Interestingly, one exhaustive study of the 57 species in the family Balsaminaceae, found similar results to ours (Vandelook *et al*., 2019), reporting that the total AA concentration and the amount of Met both exhibited phylogenetic signal while the profile of AAs produced by a plant species did not. This supports our contention that it is the AA blend which may be of more importance to pollinators. However, they found that there was no evidence that AA nectar traits were associated with pollinator group (Vandelook *et al*., 2019). By contrast, a second study found no phylogenetic signal for total AA, EAA, or NEAA concentrations (Venjakob *et al*., 2022). However, while covering more plant families, this study involved fewer (34) species, which may limit detection of significant signal for any of the traits considered (Münkemüller *et al*., 2012).

The presence or absence of phylogenetic signal does not, however, directly imply low or high pollinator‐driven selection (Abrahamczyk *et al*., 2017). A trait that shows high signal may be phylogenetically constrained, but equally, stabilising selection within closely related species could generate similar signal (Blomberg & Garland, 2002). Meanwhile, low phylogenetic signal is indicative of selective pressure, but there are multiple agents (not just pollinators), which could be driving this (Parachnowitsch *et al*., 2019).

Nevertheless, although we found that the concentration of nectar AAs may be phylogenetically constrained, our data show that the profile of EAAs in nectar is a more labile trait, which could be driven by pollinator preferences. The behavioural experiments also suggest that EAA blends may be more important to pollinators than overall EAA concentrations. For example, both the EAA blends represented by cluster B and cluster D had Met as a significant proportion of the total EAA concentration. While the blend of EAAs in cluster D stimulated feeding behaviour of bumblebees, the same was not true for cluster B. Furthermore, varying the total concentration of the EAA blend in cluster B across several orders of magnitude had little impact on feeding preference. Previous studies of AA preferences of pollinators have overwhelmingly focussed on individual AAs (Inouye & Waller, 1984; Carter *et al*., 2006; Bertazzini & Forlani, 2016; Hendriksma & Shafir, 2016), while studies testing preferences for AA blends are few, particularly for bees (e.g. Stabler *et al*., 2015). Individual AAs can either be aversive to bees or promote feeding (Inouye & Waller, 1984; Kim & Smith, 2000; Bertazzini & Forlani, 2016; Hendriksma & Shafir, 2016; Bogo *et al*., 2024), suggesting differential effects of individual AAs on reward valuation. Furthermore, not all AAs are detectable or discernible via chemotactile sensation in bumblebees (Ruedenauer *et al*., 2019) and wasps (Mattiacci *et al*., 2023). Taken together, this suggests that the relative blend of AAs is likely driving the feeding effects we observed with our EAA mixtures, rather than the presence or absence of individual AAs.

While including EAAs increased the amount of solution consumed, the bees did not avoid or prefer the solutions containing EAAs, regardless of their concentration, when they were tested over the μM range (Fig. 4a). These data indicate that bees cannot taste low concentrations of EAAs, but that they have postingestive mechanisms for detecting them in food (Paoli *et al*., 2014; Stabler *et al*., 2015). EAAs such as the branched‐chain AAs, Leu, Ile, and Val, are known to drive feeding postingestively in mammals (Solon‐Biet *et al*., 2019), insects (Piper *et al*., 2017), and in bees (Stabler *et al*., 2015). Relatively high concentrations of free EAAs can be aversive or even toxic to bees (Paoli *et al*., 2014). For this reason, we predict that although the concentrations of these EAAs are low in nectar, they are in a range that is not aversive but still valuable to pollinators as a source of essential nutrients. We expect this postingestive effect takes a longer time (s) for the brain to process than taste cues (ms) (Simcock *et al*., 2018).

Two individual EAAs of note were Met and Phe. Across four of the six nectar clusters, one of these two compounds formed a dominant component of the EAA blend. This occurs repeatedly across all the families we sampled. This pattern was particularly obvious for Phe, as eight out of the 13 families sampled had at least one species with Phe as the dominant EAA. Phe has previously been reported as a phagostimulant for honeybees (Hendriksma & Shafir, 2016) and other insects (Dethier, 1976). It is also a dominant feature of the nectar of many bee‐visited plants, especially in the Lamiaceae (Petanidou *et al*., 2006).

Proportionally high concentrations of Met in nectar occurred in seven out of the eight Plantaginaceae species in our dataset. Met is particularly important as a dietary component for egg production in insects (Grandison *et al*., 2009; Lee *et al*., 2014). However, high concentrations of Met can be toxic and reduce lifespan (Manoukas, 1981; Grandison *et al*., 2009; Lee *et al*., 2014). Dietary Met could be particularly important for bees: Free Met is needed for genome methylation especially during development, and methylation is a mechanism important for caste differentiation and other processes in eusocial bees, as *c*. 35% of the bee genome is methylated (Foret *et al*., 2009). This is in contrast to other insects like Drosophila where only *c*. 1% is methylated (Takayama *et al*., 2014). Owing to the low absolute concentrations of any AAs in nectar, any implications for bee nutrition are likely to be less important than effects on feeding behaviour. This is because bees rely on pollen for most of their protein and AA requirements (Wright *et al*., 2018).

Previous work has found that, often, one or two AAs occur at much higher concentrations than the remaining AA complement of nectar (e.g., Phe (Petanidou *et al*., 2006), Ala (Göttlinger & Lohaus, 2024), and Pro (Kaczorowski *et al*., 2005)). In comparison with all the other AAs in our dataset, the NEAA Pro was the most common. Pro was present in 87% of species at > 0.001 μM and highly abundant. Pro is clearly important for pollinators: It forms, by far, the dominant component of haemolymph AAs in honeybees (Crailsheim & Leonhard, 1997) and bumblebees (Stabler *et al*., 2015). It also can power insect flight (Bursell, 1975); though in bees, the importance of this role is disputed (Micheu *et al*., 2000; Carter *et al*., 2006; Syromyatnikov *et al*., 2013; Stec *et al*., 2021). Interestingly, in our behavioural experiments, the bees were very sensitive to the presence of Pro in the nectar solutions, as they detected and avoided quite low (10 μM) concentrations of Pro. Curiously, their aversion to Pro was reduced as its concentration increased from 10 to 1000 μM in nectar.

Plants are likely to have mechanisms for the active regulation of the quantities of AAs in nectar, but only a few studies have tested this. For example, in bromeliads, the AA concentrations in leaves and nectaries were much greater than in nectar (Göttlinger & Lohaus, 2022). This regulation may not be complete though, as several studies have shown that experimental addition of fertiliser leads to higher nectar AA concentrations (Gardener & Gillman, 2001b; Gijbels *et al*., 2015a). However, regulation of AA composition at the level of the nectaries also appears to extend to individual AAs or the AA profile (Lohaus & Schwerdtfeger, 2014; Bertazzini & Forlani, 2016; Göttlinger & Lohaus, 2024). Particularly, striking is an example of two groups of species from the bromeliad genus *Pitcarnia*. Both groups have high Ala concentrations in phloem tissue, but species in one group have high Ala concentrations in the nectar as well, whereas the others do not (Göttlinger & Lohaus, 2024).

Differences in AA composition between nectar and other plant tissues may occur through mechanisms other than active regulation by the plant. For example, postsecretory modification of AA composition can occur by contamination with pollen from the same plant, and as a result of pollinator visits, which may introduce pollen from other plants and microbes (Bogo *et al*., 2021). More generally microbes are common in nectar (Herrera *et al*., 2009; Fridman *et al*., 2012) and their presence can alter AA composition (Peay *et al*., 2012). The final AA profile of nectar experienced by pollinators is therefore likely to be a complex result of multiple internal and external factors.

Our data show how pollinators could play a role in shaping the presence and concentration of AAs in nectar. While there is good evidence that carbohydrate composition correlates with pollinator guild (Abrahamczyk *et al*., 2017 and others), such data are currently lacking for AAs. Here, we present evidence that from the perspective of potential pollinator selection, EAA profile is more important than EAA concentration. We found that EAA profile was not phylogenetically constrained and that pollinator feeding behaviour varied more with changes in EAA profile than across a wide range of EAA concentrations. We suggest that future research further investigates the role of EAA profiles in determining the extent to which nectar AAs drive plant–pollinator coevolution.

## Competing interests

None declared.

## Author contributions

RHP and GAW conceptualised the study. RHP and GAW developed the methodology. RHP and AEMR conducted the formal analysis. EFP, RHP, KW and AEMR carried out the investigation. RHP and AEMR performed the data curation. RHP and JGP prepared the visualisations. GAW, RHP and JGP wrote the original draft of the manuscript. GAW and RHP acquired the funding. All authors contributed to the reviewing and editing of the manuscript.

## Disclaimer

The New Phytologist Foundation remains neutral with regard to jurisdictional claims in maps and in any institutional affiliations.

## Supporting information


**Fig. S1** Phylogenetic trees highlighting total NEAA and EAA concentrations, and glucose and sucrose proportions across species.
**Fig. S2** Mortality curves for bumblebee preference assays.

**Table S1** Nectar sugar and amino acid concentration summary.
**Table S2** Test solutions from the preference assays.
**Table S3** Phylogenetic analysis statistics.
Please note: Wiley is not responsible for the content or functionality of any Supporting Information supplied by the authors. Any queries (other than missing material) should be directed to the *New Phytologist* Central Office.

## Data Availability

All data and code are available at doi: 10.25446/oxford.27867669.v1.
