# Supplementary material for: Do pollinators play a role in shaping the essential amino acids found in nectar?
Source: New Phytol. 2025 Jan 2;245(5):2279–91. doi: 10.1111/nph.20356 (PMC11798901; doi:10.1111/nph.20356)
Supplement: Supplementary file 1 — Fig. S1 Phylogenetic trees highlighting total NEAA and EAA concentrations, and glucose and sucrose proportions across species. Fig. S2 Mortality curves for bumblebee preference assays. [file NPH-245-2279-s002.pdf]

New Phytologist Supporting Information

Article Title: ‘Do pollinators play a role in shaping the essential amino acids found in nectar?’

Authors: Parkinson, R. H., Power, E. F., Walter, K., McDermott-Roberts, A. E., Pattrick, J. G., Wright, G. A.

Article acceptance date: 27 November 2024

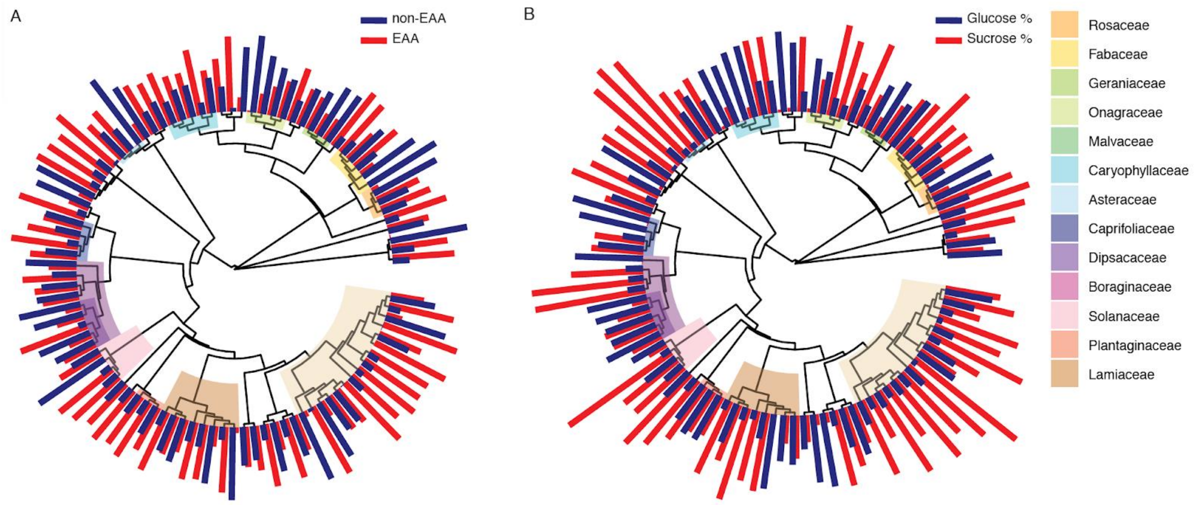

SI Figure 1: Phylogenetic trees constructed with 96 species from our nectar dataset. Red and blue bars display the total concentrations of NEAAs and EAAs (A) and the proportions of glucose and sucrose (B) across the dataset, with families highlighted on each tree where the number of species is >2.

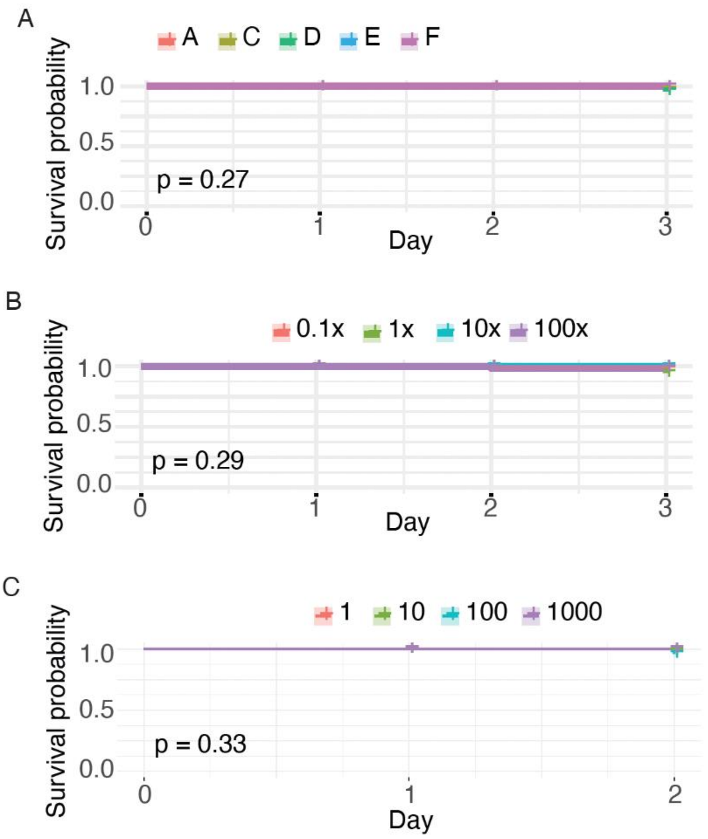

SI Figure 2: Kaplan-Meier mortality curves for the three bumblebee preference assays: nectar relevant concentrations of cluster A, C, D, E, and F nectar (A), a concentration gradient of cluster B nectar profile (B) and the proline preference assay (C). There was minimal mortality across any of the assays, and no significant difference between groups (n = 75 bees per group). Exact p-values shown on each plot.

SI Table 1: Summary of the mean and standard errors of concentrations of sugars and amino acids across samples for each species.

*** Table is attached (too large to display) ***

SI Table 2: Solutions used in the three preference assays. Amino acids are reported in micromolar (µM), and sugars in sucrose-equivalent molarity (se-M).

	Test solution	His µM	Thr µM	Arg µM	Val µM	Met µM	Trp µM	Phe µM	Ile µM	Leu µM	Lys µM	Pro µM	Suc se-M	Fruc se-M	Gluc se-M
**Assay 1**	A	0.00	0.00	0.00	0.00	0.04	0.00	0.01	0.00	0.00	0.04		0.21	0.24	0.21
	C	0.75	1.00	2.15	1.53	6.40	0.44	3.22	0.68	1.09	4.15		0.25	0.28	0.18
	D	0.13	0.05	0.07	0.11	0.49	0.00	0.19	0.11	0.26	0.12		0.33	0.28	0.19
	E	0.01	0.28	1.15	0.43	0.01	0.01	0.14	0.14	0.13	0.04		0.18	0.25	0.15
	F	0.69	0.32	0.68	1.17	0.58	0.00	7.45	0.50	0.49	0.22		0.29	0.30	0.14
	G	0.55	0.23	0.75	3.06	0.87	0.00	0.91	0.61	0.76	0.49		0.26	0.25	0.16
**Assay 2**	1 µM	0.04	0.05	0.10	0.07	0.30	0.02	0.15	0.03	0.05	0.19		0.25	0.28	0.18
	10 µM	0.35	0.47	1.00	0.71	2.99	0.21	1.50	0.32	0.51	1.94		0.25	0.28	0.18
	100 µM	3.52	4.67	10.05	7.14	29.89	2.06	15.03	3.19	5.07	19.39		0.25	0.28	0.18
	1000 µM	35.2	46.7	100.5	71.4	298.9	20.6	150.3	31.89	50.68	194.0		0.25	0.28	0.18
**Assay 3**	1 µM											1		0.50	0.50
	10 µM											10		0.50	0.50
	100 µM											100		0.50	0.50
	1000 µM											1000		0.50	0.50

SI Table 3: Results from phylogenetic analysis using Blomberg's K and Pagel's Lambda. The EAA profiles (t-SNE dimensions), proportions of individual EAAs and proline, total concentrations of EAAs, NEAAs, and sugars, and proportions of sugars were tested. Significant p-values are highlighted in orange.

Nectar variable	Blomberg's K		Pagel's Lambda	
	stat	p-value	stat	p-value
t-SNE Dim 1	0.066	0.209	0.000	1.000
t-SNE Dim 2	0.051	0.682	0.000	1.000
His %	0.051	0.566	0.000	1.000
Thr %	0.031	0.978	0.000	1.000
Arg %	0.056	0.475	0.000	1.000
Val %	0.048	0.771	0.000	1.000
Met %	0.099	0.060	0.577	0.042
Trp %	0.034	0.836	0.000	1.000
Phe %	0.094	0.038	0.044	0.530
Ile %	0.062	0.336	0.000	1.000
Leu %	0.042	0.744	0.000	1.000
Lys %	0.046	0.798	0.000	1.000
Pro %	0.100	0.018	0.035	0.669
EAA (μM)	0.440	0.009	0.913	0.001
NEAA (μM)	0.137	0.018	0.346	0.384
Sugars (M)	0.073	0.129	0.000	1.000
Sucrose %	0.084	0.024	0.026	0.695
Fructose %	0.071	0.136	0.000	1.000
Glucose %	0.080	0.036	0.067	0.420
